# Simulation of Premovement Active Surveillance Protocols for Moving Finishing Pigs to a Harvest Facility from a Control Area during an Outbreak of African Swine Fever in the United States

**DOI:** 10.1155/2024/6657600

**Published:** 2024-07-15

**Authors:** Peter J. Bonney, Sasidhar Malladi, Amos Ssematimba, Kathleen C. O'Hara, Marta D. Remmenga, Michelle Farr, Mickey Leonard, Catherine Y. Alexander, Benjamin Blair, Sylvia Wanzala Martin, Marie R. Culhane, Cesar A. Corzo

**Affiliations:** ^1^ Secure Food Systems Team University of Minnesota, St. Paul, MN 55108, USA; ^2^ Department of Mathematics Faculty of Science Gulu University, Gulu, Uganda; ^3^ US Department of Agriculture Animal and Plant Health Inspection Service Veterinary Services Strategy and Policy Center for Epidemiology and Animal Health, Fort Collins, CO, USA

## Abstract

Movement restrictions are a critical component of response plans for an African swine fever (ASF) outbreak in the United States. These restrictions are likely to include requiring permits to move animals and products within, into, and out of 5-km control areas (CAs) established around confirmed positive farms. For quarantined finishing farms located within a CA, diagnostic testing is an expected criterion for receival of a permit to move pigs to a harvest facility or removal of quarantine. A stochastic disease transmission and active surveillance model were used to evaluate premovement active surveillance protocols varying by the number of samples and timing of sample collection before movement. Surveillance protocol scenarios were evaluated for several different sampling prioritization schemes; virus strains of medium or high virulence; barn sizes of 1,200, 2,400, and 4,800 pigs; and farms with average to high mortality and morbidity during routine production. Surveillance protocols that included prioritization schemes targeting dead pigs and pigs with clinical signs resulted in the highest probabilities of detection and the lowest numbers of infectious pigs at the time of movement in barns that went undetected. There was some evidence that targeting sick pigs prior to dead pigs may be more effective for moderately virulent strains. However, in most scenarios, including all highly virulent strain scenarios and moderately virulent strain scenarios in barn sizes of 1,200 with average farm performance, prioritization of dead versus sick pigs first did not have a large impact on the predicted outcomes. Increasing sample sizes improved outcomes, though only marginal gains were achieved once the available dead and sick were sampled. Predicted outcomes may be further improved by sampling the available dead and sick pigs in a barn across multiple days, though the associated increase in the probability of detection was minor.

## 1. Introduction

African swine fever (ASF) is a reportable disease of high concern for the swine industry worldwide. As of this writing, there is no well-established, commercially available vaccine or treatment for ASF. An introduction of African swine fever virus (ASFV) into a barn can severely hamper production, especially due to high mortality, which can approach 100% for highly virulent strains and in naïve populations [1]. Following the 2007 introduction of ASFV into the country of Georgia, the virus has steadily spread to numerous countries in Europe and Asia [2]. In 2021, ASF was detected in the western hemisphere for the first time in over 40 years, initially in the Dominican Republic and then confirmed in Haiti shortly thereafter [3].

In the United States (US), control measures for a potential ASF outbreak include quarantines, movement controls, enhanced biosecurity, epidemiologic tracing, and stamping out [4]. Herds confirmed to have ASF would be depopulated as soon as possible. A 5-km control area (CA), consisting of a 3-km infected zone and 2-km buffer zone, would be established around any confirmed positive premises. An additional 5-km surveillance zone would extend beyond the CA. Movement of animals or products within, into, and out of the CA would typically require a permit. The criteria for the permit could vary depending on the risk related to the movement in question and may require additional actions such as biosecurity and active surveillance using diagnostic testing.

Movement of finishing pigs to a harvest facility from a farm located within a CA is expected to require a permit. Negative diagnostic test results from samples collected in the days immediately prior to the scheduled movement would likely be part of the permit criteria. In this analysis, a stochastic within-barn ASFV transmission and active surveillance model were used to evaluate premovement active surveillance protocols at the barn level to be performed prior to the movement of finishing pigs to a harvest facility.

Mathematical modeling has been used extensively to evaluate control measures for an outbreak of ASF [5]. However, the focus of these modeling studies has been on response measures such as size and duration of CA-type zones established around confirmed positive farms; culling of farms within a certain distance of confirmed positive farms; varying intensities of regular active surveillance of farms located within CA-type zones; and mortality triggers for initial detection of ASF within commercial pig farms [6, 7, 8, 9, 10, 11, 12]. Movements within and out of the zones established around confirmed positive premises were often assumed to be highly restricted or prohibited [6, 7, 8, 10, 11]. Sykes et al. [13] modeled ASF control in the US, including movement permits for farms located within a CA but modeled premovement active surveillance approximately and did not interrogate the impact of different premovement active surveillance protocols on their results.

The premovement active surveillance protocols included in this analysis consist of both a mortality trigger and testing of tissue specimens and/or blood from individual pigs for ASFV particles via real-time, semiquantitative, or quantitative polymerase chain reaction (qPCR) assays. The active surveillance protocols were evaluated at the barn level based on the probability of detection prior to movement and mean number of infectious pigs at the time of movement in barns where ASF infections went undetected. These outcomes were estimated using varying combinations of many input parameters such as different prioritization schemes for sampling dead and sick pigs; a highly virulent and moderately virulent ASFV strain; different barn sizes; and various rates of mortality and morbidity during routine production. The aim of this work was to provide recommendations for active surveillance to optimize probability of detection and limit the number of infectious pigs moved during an ASF outbreak in the US.

## 2. Materials and Methods

### 2.1. Stochastic Within-Barn ASFV Transmission Model

The spread of ASFV within a finisher barn was simulated using the heterogeneous transmission model described in Ssematimba et al. [14]. The heterogeneous transmission model considers two modes of transmission for virus spread, direct contact of pigs within and between pens and fomite transmission that can jump between pens located in different barns or rooms. The model tracks the movement of individual pigs between disease states at 0.01-day time steps (Δ*t*). The disease state compartments include the standard compartments in an SEIR model, susceptible, exposed, latently infected, infectious, and removed (i.e., dead or recovered). Additional compartments were implemented for simulating active surveillance of viremic pigs and pigs with clinical signs.

Within-barn ASFV transmission was simulated starting with a single latently infected pig randomly placed in a pen and room. The number of susceptible pigs in a pen *k* located in room *r* in time step *t* that become infected by *t*+Δ*t* was simulated from a binomial distribution. The probability that a susceptible pig in a nonedge pen *k* becomes infected by *t*+Δ*t* is given by the following expression:

(1) $$\begin{array}{c} 1-e^{-\left(\beta_{within\;direct}\frac{I_{r,k}}{N_{r,k}}+\beta_{between\;direct}\frac{I_{r,k-1}+I_{r,k+1}}{N_{r,k-1}+N_{r,k+1}}+\beta_{dist\;ind}\frac{\sum_{i}I_{r,i}}{\sum_{i}N_{r,i}}+\beta_{room}\frac{\sum_{m\neq r}\sum_{i}I_{m,i}}{\sum_{m\neq r}\sum_{i}N_{m,i}}\right)\Delta t}. \end{array}$$

**Figure 1:**
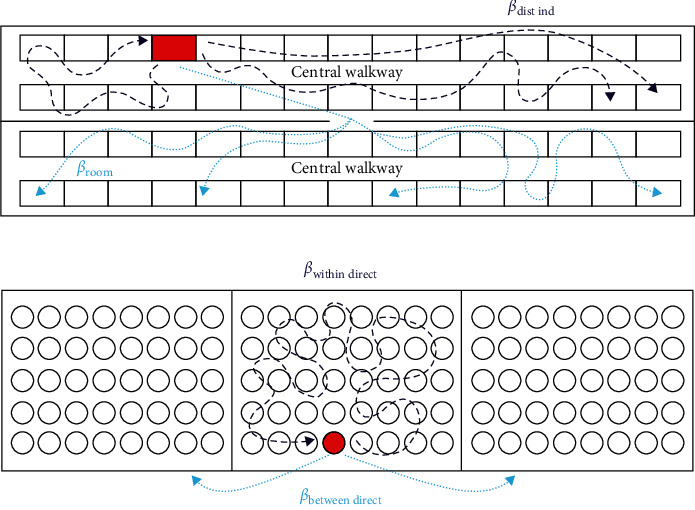
Representation of the transmission mechanisms implemented within the ASFV transmission model. (a) Distance-independent transmission (*β*_**dist** **ind**_, dark blue dashed line) to pens in the same room as an infectious pen (marked in red) and transmission to pens in a different room within the same barn (*β*_**room**_, light blue dashed line). (b) Transmission between pigs in the same pen as an infectious pig (marked in red) due to direct contacts (*β*_**within** **direct**_, dark blue dashed line) and transmission to pigs in adjacent pens due to direct contacts (*β*_**between** **direct**_, light blue dotted line).

The following definitions for the transmission terms were used: *β*_*within* *direct*_ referred to within-pen transmission due to direct contact with pigs in the same pens; *β*_*between* *direct*_ referred to between-pen transmission due to direct contact with pigs in adjacent pens (nose to nose contact); *β*_*dist* *ind*_ referred to transmission due to distance-independent mechanisms (e.g., fomites and people) within a room assumed to occur at a constant frequency; and *β*_*room*_ referred to transmission mechanisms that occur between rooms in the same barn. See Figure 1 for a visualization of the transmission associated with *β*_*within* *direct*_, *β*_*between* *direct*_, *β*_*dist* *ind*_, and *β*_*room*_.

The terms *I*_*r*,*k*_ and *N*_*r*,*k*_ referred to the number of infectious and alive pigs, respectively, in room *r* and pen *k*. Similarly, *I*_*r*,*k*−1_ and *I*_*r*,*k*+1_ were the number of infectious pigs in the pens adjacent to pen *k* in room *r*, while *N*_*r*,*k*−1_ and *N*_*r*,*k*+1_ were the total number of pigs in the adjacent pens. The term ∑_*i*_*I*_*r*,*i*_ was the total number of infectious pigs in each pen *i* in room *r*, and $\sum_{i}N_{r,i}$ was the total number of living pigs in each pen *i* in room *r*. Last of all, $\sum_{m\neq r}\sum_{i}I_{m,i}$ was the total number of infectious pigs in each pen *i* in each room *m* (excluding room *r*), while $\sum_{m\neq r}\sum_{i}N_{m,i}$ was the total number of living pigs in each pen *i* in each room *m* (excluding room *r*).

Transmission rate estimates for ASF are commonly given in the scientific literature in terms of the contacts occurring exclusively within pens (*β*_*w*_) and exclusively between pens (*β*_*b*_) [15, 16]. The *β*_*w*_ and *β*_*b*_ parameters must be adapted for use in the Ssematimba et al. [14] approach since within- and between-pen transmission in Ssematimba et al. [14] were divided into direct and distance-independent pathways. The *β*_*within* *direct*_, *β*_*between* *direct*_, and *β*_*dist* *ind*_ formulation for within-room transmission were related to the *β*_*w*_ and *β*_*b*_ formulation in the following way. Let the mean proportion of between-pen contacts due to distance-independent pathways be *θ*. Then,

(2) $$\begin{array}{c} \beta_{between\;direct}=\beta_{b}\left(1-\theta \right) \end{array}$$

(3) $$\begin{array}{c} \beta_{dist\;ind}=\beta_{dist\;ind}\theta \frac{\sum_{i}N_{i}}{\sum_{i\neq k}N_{i}} \end{array}$$

(4) $$\begin{array}{c} \beta_{within\;direct}=\beta_{w}-\beta_{dist\;ind}\frac{N_{k}}{\sum_{i}N_{i}} \end{array}$$

For values of *θ* close to 1, transmission of ASFV approximated distance-independent homogeneous spread. As *θ* approached 0, transmission became more clustered within and around the pen housing an infectious pig. For the derivation of these equations and more discussion, see Ssematimba et al. [14].

The number of simulation time periods a pig remained in each disease state was simulated from the probability distributions described in Tables 1 and 2. Once a pig became infected, it transitioned into the latently infected state. Pigs in the latently infected state transitioned into the infectious state prior to either dying or recovering. Infected pigs transitioned into the viremic state prior to or at the time of the transition into the infectious state. Pigs transitioned out of the viremic state once recovered or dead. Pigs developed clinical signs at the time of or after the transition into the infectious state. All infected pigs were assumed to develop clinical signs. Pigs that recovered could transition out of the clinical signs' state prior to transitioning into the recovered state. Pigs that died transitioned out of the clinical signs' state at the time of death. Once an infected pig transitioned into the dead or recovered state, it remained in that state for the remainder of the simulation; recovered pigs were no longer susceptible.

The heterogeneous transmission model was coded using the C programming language and R statistical software [21, 22]. The model ran for 10,000 iterations (less than 2% margin of error in probability of detection estimates), and the number of pigs in each disease state over time was stored at 0.25-day time intervals.

### 2.2. Input Parameters for the Stochastic Within-Barn ASFV Transmission Model

Two ASFV strain scenarios were evaluated, a moderately virulent strain scenario and a highly virulent strain scenario. The transmission model input parameters are given in Table 1 for the moderately virulent strain scenario and in Table 2 for the highly virulent strain scenario.

The disease state distributions for the moderately virulent strain scenario were determined as follows. The distribution for the onset of viremia for the moderately virulent strain scenario was estimated from experimental data reported in de Carvalho Ferreira et al. [17] involving moderately virulent Malta 78 and Netherlands 86 genotype I ASFV strains. In the de Carvalho Ferreira et al. [17] experiment, five out of 10 total 12-week-old pigs were inoculated per ASFV strain and dose scenario. Each group of 10 pigs was placed together in the same room. The remaining disease state distributions (length of the latent and infectious period for pigs that recover or die, time to onset of clinical signs, and duration of clinical signs) were retrieved from Malladi et al. [12], who also estimated the distributions from the de Carvalho Ferreira [17] experimental data.

The following describes the transmission rate parameters used in the moderately virulent strain scenario. The within-pen transmission rate (*β*_*w*_) was estimated by Malladi et al. [12]. The between-pen transmission rate (*β*_*b*_) was taken from Guinat et al. [15], who estimated it from an experiment by Guinat et al. [23] involving a highly virulent Georgia 2007/1 genotype II ASFV strain. In the Guinat et al. [23] experiment, 40 total 7-week-old female large white pigs were placed in different configurations of inoculated and contact pigs. In room A, five inoculated pigs were placed with five contact pigs; in rooms B and C, four inoculated pigs were placed with four contact pigs in the same pen with four other contact pigs in an adjacent pen; and three inoculated pigs were placed with three contact pigs in room D [23]. The proportion of between-pen spread in a room due to distance-independent pathways (*θ*) was set to 0.05 based on reports of clustered spread of ASFV between pens [18, 19]. The *β*_*w*_ and *β*_*b*_ parameters were adapted to the Ssematimba et al. [14] approach with *β*_*within* *direct*_, *β*_*between* *direct*_, and *β*_*dist* *ind*_ as described above. As of writing, a between-room transmission rate (*β*_*room*_) had not been estimated from field or experimental data for ASF. As such, the distribution was based on expert opinion. However, the authors note that the distribution is comparable to that used in Faverjon et al. [9], also based on expert opinion.

The disease state distributions for the highly virulent strain scenario were estimated from an experiment by Yamada et al. [20] involving pigs inoculated with the highly virulent Armenia 07 genotype II ASFV strain. Data selected from Yamada et al. [20] included three pigs inoculated with 1 mL of 0.1 *HAD*_50/*ml*_ of virus inoculum, three pigs inoculated with 10^1^ *HAD*_50/*ml*_ of virus inoculum, and two trials where one pig was inoculated with 10^3^ *HAD*_50/*ml*_ of virus inoculum and placed with two contact pigs. All pigs were 8-week-old Landrace—Large White—Duroc pigs. Parameters were estimated using a maximum likelihood method. The within- and between-pen transmission rates for the highly virulent strain scenario were taken from Hu et al. [16], who estimated the parameters from the Guinat et al. [23] experiment. The proportion of between-pen spread due to distance-independent pathways (*θ*) was set to 0.05 to capture clustering of disease spread. The between-room transmission rate was based on expert opinion.

ASFV transmission was simulated for three barn sizes. The baseline scenario consisted of a single room with 1,200 pigs. Within that room, pens were arranged in two rows with 15 pens per row and 40 pigs per pen. The two rows were separated by a central walkway. Pens were considered adjacent if they touched pen dividers in the same row. The second barn size consisted of two rooms with a structure identical to the one just described, that is, each room contained 1,200 pigs with two rows of 15 pens each separated by a walkway for a total of 2,400 pigs (Figure 1(a)). The third barn size consisted of four rooms of this type for a total of 4,800 pigs.

### 2.3. Active Surveillance Simulation Model

In the active surveillance model, we simulated the application of a mortality trigger as well as qPCR testing of tissue specimens from dead pigs or blood from pigs with clinical signs and apparently healthy pigs (i.e., pigs without clinical signs). Input parameters for the active surveillance model are given in Table 3. The total number of pigs in the dead pig subpopulation in the barn was broken down into two groups, pigs that died due to ASF and pigs that died as part of routine production-limiting, that is, endemic or preexisting, diseases. Similarly, the subpopulation of pigs with clinical signs was divided into pigs that had clinical signs due to ASF and pigs that had clinical signs consistent with ASF but due to other causes. Clinical signs ranged from mild (low-grade fever, inactivity, and anorexia) to severe (hyperpnea, vomiting, and bloody diarrhea) [1]. The subpopulation of apparently healthy pigs was broken down into pigs infected with ASFV and viremic (but displaying no clinical signs) and all other pigs with no clinical signs.

The number of dead pigs due to ASF over time was taken from the disease transmission model output. The mortality not due to ASF was simulated based on weekly mortality data from 248 finishing pig herds from four pig farming systems in North America during routine production. The weekly mortality was approximately exponentially distributed with a mean rate of three per 1,000. We also considered a high mortality rate scenario focusing on the top 5% of the weekly mortality in the data. The top 5% of the weekly mortality was approximately exponentially distributed with a mean rate of 14 per 1,000.

The number of pigs with clinical signs due to ASF over time was also taken from the disease transmission model output. The number of pigs with clinical signs not due to ASF during routine production was simulated from distributions parameterized based on input from North American swine industry expert opinion [12]. We considered average and high scenarios for the proportion of pigs with clinical signs during routine production. The proportion of viremic pigs among the population with no clinical signs was estimated from disease transmission model output.

The active surveillance model was flexible in terms of the number of pigs to be sampled for qPCR testing and the sampling prioritization scheme. For example, suppose we wanted to sample dead pigs first, then pigs with clinical signs, and then pigs without clinical signs. The application of the active surveillance protocol was simulated as follows. Tissue specimens from dead pigs were pooled separately from the blood of sick and apparently healthy pigs to maintain specimen integrity. Blood was pooled regardless of health status, meaning blood from apparently healthy pigs was pooled with the sick pig samples. A pooled sample, therefore, consisted of either five tissue specimens or five blood vials.

On a sampling day, the pigs that died over the previous 24 hr were sampled until either all dead pigs had been sampled or the target sample size was reached. A hypergeometric distribution was used to simulate the number of dead pigs due to ASF (out of all dead pigs from all causes) included in each pooled sample of five. If there was at least one dead pig due to ASF in the pooled sample, then that sample tested positive by qPCR according to the test sensitivity, set here to 0.95 based on Pikalo et al. [24].

If the target sample size had not been reached following the sampling of dead pigs, then blood was collected from pigs with clinical signs. Not all pigs with mild clinical signs may be noticed by workers in the barn. To account for this possibility, the probability of observing a pig with clinical signs was set to 0.85 based on input from a workgroup consisting of US swine industry veterinarians, state and government regulators, and academicians (ASF Stakeholder Workgroup, personal communication, November 2022). The sick pigs observed at the time of active surveillance were sampled until either all sick pigs had been sampled or the target sample size had been reached. The sick pig specimens were divided equally between the remaining pools of five. The number of pigs sampled with clinical signs due to ASF included in each pool was simulated from a hypergeometric distribution.

If the target sample size had still not been reached, blood was collected from a random selection of apparently healthy pigs. The number of viremic apparently healthy pigs included in each pooled sample was simulated from a binomial distribution. The probability that a pig was viremic was set to the prevalence of viremic pigs among pigs with no clinical signs as determined from the ASFV transmission model output. If a pooled sample contained at least one pig with clinical signs due to ASF or at least one viremic pig, then the sample tested positive by qPCR with probability equal to the test sensitivity of 0.95.

A similar process was used to simulate the prioritization sampling schemes of sick, then dead, then apparently healthy sampling, and random sampling of living pigs. ASFV was detected in the herd if at least one positive qPCR result was obtained or the total mortality over the past 24 hr exceeded the trigger threshold on a day. We used a mortality trigger threshold of five dead per 1,000 based on the analysis from Malladi et al. [12]. The active surveillance model was coded in R statistical software [22].

### 2.4. Active Surveillance Protocol Scenarios

Eight barn-level active surveillance protocols specifically designed for testing of finishing pigs prior to being sent from a CA to a harvest facility were evaluated. The active surveillance protocols varied by the number of individual pig specimens collected for testing by qPCR and whether specimens were collected 24 and 48 hr prior to movement or only 24 hr prior to movement. A description of the active surveillance protocols can be found in Tables 4 and 5.

In addition to specimens collected for qPCR testing, a mortality trigger was applied for each protocol. Mortality exceeding five dead pigs per 1,000 in the barn on a day would prompt further investigation. It was assumed that this investigation would result in detection of ASF with 100% certainty. Thus, mortality exceeding five per 1,000 was considered as sufficient for or equivalent to detection.

The active surveillance protocols were evaluated for scenarios varying by sampling prioritization scheme, barn size, average or high mortality and morbidity during routine production, and ASFV strain. The sampling prioritization schemes included:

1. Collecting tissue specimens from dead pigs first, then blood from pigs with clinical signs, and then blood from apparently healthy pigs as needed until the target sample size was reached
2. Collecting blood from pigs with clinical signs, then tissue specimens from dead pigs, followed by blood from apparently healthy pigs
3. Collecting tissue specimens from up to five dead pigs, then blood from pigs with clinical signs, and then blood from apparently healthy pigs
4. Collecting blood from randomly selected living pigs with or without clinical signs

The active surveillance protocols were evaluated based on the probability of detecting ASFV in the barn prior to movement of pigs off the premises and the number of infectious pigs in the barn at the time of movement if ASFV went undetected. These metrics were estimated considering different times of virus exposure prior to the scheduled movement day. A total of 30 days prior to movement was chosen as a cutoff for the time of virus exposure based on Malladi et al. [12], who estimated 30 days to be the mean time to detection of ASFV in finisher pig barns for a slow spreading, moderately virulent strain.

## 3. Results

### 3.1. Prevalence of Pigs with Detectable ASFV over Time Post Virus Exposure

**Figure 2:**
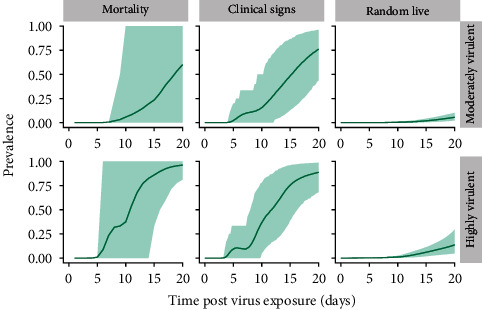
Mean (solid line) and 95% P.I. (shaded area) for the prevalence of pigs with detectable ASFV in different subpopulations for 1,200 head barns with average routine mortality and morbidity.

The prevalence of pigs with detectable ASFV among the subpopulations of pigs with clinical signs, dead pigs, and living pigs (pigs with or without clinical signs) simulated for the baseline scenario of 1,200 head barns with average routine mortality and morbidity is given in Figure 2 for the moderately and highly virulent ASFV strain scenarios.

Prevalence of detectable pigs increased first in the population of pigs with clinical signs. A 5% threshold for the mean prevalence was exceeded 5.75 days post exposure (DPE) in the moderately virulent strain scenario and 4.5 DPE in the highly virulent strain scenario. In comparison, the prevalence of detectable pigs in the dead pig subpopulation exceeded a 5% threshold 10 DPE in the moderately virulent strain scenario and 6 DPE for the highly virulent strain scenario. There was substantial variability in the prevalence of detectable pigs from within the dead pig subpopulation. This was especially true in the moderately virulent strain scenario, where the 95% P.I. ranged from 0% to 100% prevalence even up to 20 DPE. The prevalence of detectable pigs increased much more slowly in the subpopulation of living pigs (including sick and apparently healthy pigs) than in the daily mortality subpopulation and the subpopulation of pigs with clinical signs. In the living pig subpopulation, a 5% threshold for the mean prevalence was first exceeded 19.25 DPE in the moderately virulent strain scenario and 14.25 DPE in the highly virulent strain scenario. The prevalence in the different subpopulations increased more quickly in the highly virulent strain scenario relative to the moderately virulent strain scenario.

### 3.2. Predicted Active Surveillance Outcomes for 1,200 head, Averagely Performing Barns

Table 4 summarizes the probability of detection and mean number of infectious pigs at the time of movement in barns where ASF went undetected considering a barn size of 1,200 pigs with average rates of mortality and morbidity during routine production.

Regardless of the protocol evaluated or ASFV strain, the probability of detection was substantially higher when sick and dead pigs were targeted for sampling relative to randomly sampling living pigs. Furthermore, targeting sick and dead pigs was estimated to result in lower numbers of infectious pigs at the time of movement in barns that go undetected. Results were nearly identical between the sampling prioritization schemes with sick and dead pig targeting (many estimates within the 2% margin of error resulting from the number of simulation iterations).

While the predicted outcomes improved as the sample size increased for surveillance strategies using the random live sampling prioritization scheme, for the dead and sick pig targeting schemes, sample size increases did not always result in a corresponding increase in the probability of detection. More specifically, for protocols where all samples were collected 24 hr prior to movement, there was little change in the probability of detection and mean number of infectious but undetected pigs for 1,200 pig barns once 15 individual pig samples were reached.

Dividing 30 or greater number of samples between 2 days, as in Protocol D and Protocol F, had the highest probability of detection and fewest number of infectious undetected pigs at the time of movement. Across every scheme that prioritized sick and dead pigs, sampling 15 pigs 24 and 48 hr prior to movement resulted in a higher probability of detection than a single round of testing 30, 60, or even 100 pigs 24 hr prior to movement.

### 3.3. Effect of Barn Size and Farm Performance on the Probability of ASFV Detection

**Figure 3:**
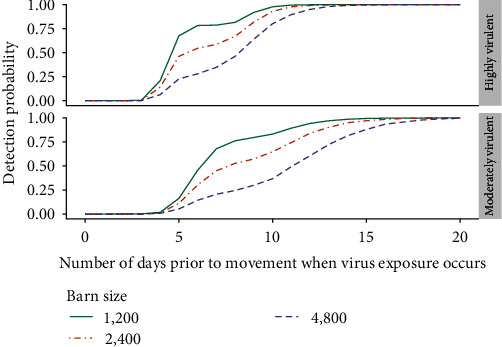
The probability of detection of ASFV by qPCR on pigs sampled in barns with sizes of 1,200, 2,400, and 4,800 pigs, for both highly and moderately virulent strain scenarios, assuming average routine mortality and morbidity. The probability of detection was estimated for times of virus exposure from 1 to 20 days prior to scheduled movement. The active surveillance protocol consisted of 15 individual pig specimens collected 24 hr prior to movement and a sampling prioritization scheme of up to five dead pigs, then pigs with clinical signs, and then apparently healthy pigs. The qPCR testing was assumed to have a 0.95 test sensitivity on sample pools of five of the same specimen type.

A comparison of the probability of detection for barn sizes of 1,200; 2,400; and 4,800 pigs is given for the moderately and highly virulent strain scenarios in Figure 3. For sampling occurring in the initial days following virus exposure or once the virus had been circulating for roughly 2 weeks or longer in the barn, the probability of detection was similar across barn sizes. However, between approximately∼ three and 13 days for the highly virulent strain scenario and four and 18 days for the moderately virulent strain scenario, an increase in barn size was associated with a decrease in the probability of detection.

**Figure 4:**
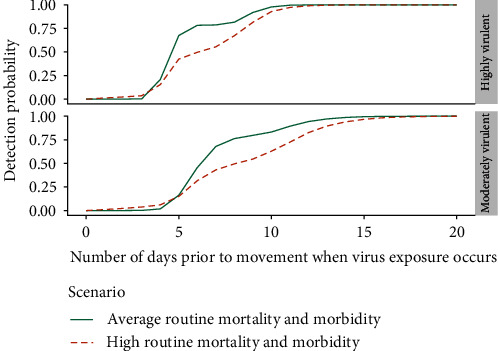
The probability of ASFV detection for average performing barns (solid line) (average mortality and morbidity during routine production) versus poorly performing barns (dashed line) (high mortality and morbidity during routine production). The probability of detection by qPCR on sample pools of five of the same specimen type was estimated for times of virus exposure ranging from 1 to 20 days prior to the scheduled movement for both highly and moderately virulent ASFV strain scenarios, assuming a barn size of 1,200 pigs. The active surveillance protocol consisted of 15 individual pig samples collected 24 hr prior to movement and a sampling prioritization of up to five dead pigs, then pigs with clinical signs, and then apparently healthy pigs.

Figure 4 again considers probability of detection against time of virus exposure prior to movements but compares farms with average routine mortality and morbidity to farms with high rates of mortality and morbidity during routine production. See Table 3 for the definition of average and high rates of routine mortality and morbidity. As can be seen in Figure 4, higher amounts of routine mortality and morbidity were associated with lower detection probabilities.

### 3.4. Predicted Active Surveillance Outcomes for 4,800 head, Poorly Performing Barns

To further explore the effect of larger barn sizes and higher routine mortality and morbidity on the probability of detection, the active surveillance protocols were also evaluated for a conservative scenario of 4,800 head barns with high routine mortality and morbidity. The prevalence of detectable pigs with ASFV among the dead pigs, pigs with clinical signs, and living pig subpopulations for the 4,800 head barns can be observed in Figure S1. The prevalence of ASFV among the different pig subpopulations was lower in Figure S1 compared to the prevalence in the subpopulations for the 1,200 head barns with average performance in Figure 2. In Figure S1, the prevalence of pigs with detectable ASFV remained higher in the dead and sick pig subpopulations relative to the living pig subpopulation.

The probability of detection and the mean number of infectious pigs at the time of movement in barns that went undetected are given in Table 5 for the 4,800 head barns. Targeting dead and sick pigs led to higher probabilities of detection and fewer infectious pigs in undetected barns at the time of movement relative to randomly sampling living pigs.

While the results were similar across the three sampling prioritization schemes with sick and dead pig targeting for the highly virulent strain, there were some differences in the moderately virulent strain scenario. In general, the sick, then dead, and then apparently healthy, prioritization schemes had the best performance in the moderately virulent strain scenario. Conversely, the dead, then sick, and then apparently healthy, prioritization schemes had the lowest probabilities of detection and the highest numbers of infectious but undetected pigs. The results of the prioritization scheme where up to five dead pigs were sampled, then sick, and then apparently healthy pigs split the difference between the results of the other two schemes with targeting of sick and dead pigs. The differences in the results between the prioritization schemes with sick and dead pig targeting were largest for the active surveillance protocols with the smallest sample sizes. These differences decreased as the sample size increased, with the results for the prioritization schemes estimated to be equivalent when 100 individual pig samples were collected.

Protocol H (100 individual pig samples collected 24 hr prior to movement) was estimated to have the highest probability of detection and the lowest number of infectious pigs in a barn that went undetected. The probability of detection increased by approximately 5%–7% with every increase in sample size number in the moderately virulent strain scenario. In the highly virulent strain scenario, this increase in the probability of detection ranged from approximately 3% to 4%. Collecting all samples 24 hr prior to movement, as opposed to splitting the collection of samples between 24 and 48 hr prior to movement, was estimated to have better outcomes for sample sizes of 30 and 60.

## 4. Discussion

In this analysis, eight premovement active surveillance protocols for detection of ASFV in a finishing pig barn located in a CA prior to the movement of pigs to a harvest facility were evaluated. Many factors, including different prioritization schemes for pig sampling, ASFV strains, barn sizes, and rates of mortality and morbidity during routine production, were considered. The performance of the active surveillance protocols was assessed based on the probability of ASFV detection and the number of infectious pigs at the time of movement in barns where the virus went undetected.

Foundational to the results is the prevalence of pigs detectable for ASFV in the different subpopulations targeted for sampling: dead pigs, pigs with clinical signs, pigs without clinical signs (i.e., apparently healthy), or living pigs (i.e., those pigs with or without clinical signs). Targeting the subpopulations for sampling that had higher prevalence of detectable pigs led to higher probabilities of detection and lower numbers of infectious pigs at the time of movement when ASFV was not detected. Since prevalence was higher in the sick and dead pig subpopulations, targeting sick and dead pigs for sampling was much more efficient than randomly sampling living pigs. Similarly, due to the low prevalence in apparently healthy pigs, sampling this subpopulation after sampling the available mortality and morbidity had limited utility. Higher routine mortality and morbidity and larger barn sizes led to lower prevalence of detectable pigs with ASF in the dead and sick pig subpopulations. Consequently, ASFV was harder to detect in the larger barn size and high routine mortality and morbidity scenarios. Larger sample sizes may need to be considered for larger pig populations, particularly when higher rates of routine mortality and morbidity are present.

Differences in dead and sick pig prioritization had the greatest impact on predicted outcomes when a fraction of the mortality and morbidity was sampled and the rate of disease transmission was slow. Hence, only in the scenario consisting of 4,800 head barns with high routine mortality and morbidity infected with a moderately virulent ASFV strain were there differences observed between sampling prioritization schemes with dead and sick pig targeting (Table 5). The prevalence of detectable pigs for ASFV was more stable in the sick pig subpopulation relative to the prevalence of detectable pigs in the dead pig subpopulation in the moderately virulent strain scenario. As a result, targeting sick pigs first, then dead pigs, and then apparently healthy pigs had the best performance for the 4,800 head barns and moderately virulent strain, arguably the scenario wherein ASF would be the most difficult to detect.

Sampling up to five dead pigs, then pigs with clinical signs, and then apparently healthy pigs may be the most robust to strain differences. By targeting dead pigs first, this prioritization scheme can take advantage of the leap in prevalence a single pig dead due to ASF can cause. However, the prevalence of detectable pigs in the daily mortality may be quite variable. Limiting the sampling of dead pigs to a maximum of five prevents the prioritization scheme from becoming overly dependent on the prevalence in mortality. Thus, prioritizing up to five dead pigs, then sick pigs, and then apparently healthy pigs balances the trade-off between higher prevalence but also greater variability in the daily mortality.

This analysis focused on highly and moderately virulent ASFV strains due their potentially greater impact on the swine industry, wide circulation throughout Asia and Europe, and proximity to the US [2, 3]. However, as Gallardo et al. [25] noted, there is evidence that increased numbers of milder infections can occur over time in areas where ASF is endemic. Indeed, Avagyan et al. described three ASFV strains from 2018 to 2020 from the South Caucasus region that produced milder clinical signs and lower mortality relative to the highly virulent Armenia 07 strain [26]. Naturally occurring attenuated strains have also been identified in China as well as low virulence strains that may have been introduced due to improper vaccination [27, 28]. Low virulence ASFV strains pose a substantial challenge for active surveillance as they are characterized by low mortality and mild clinical signs and may even result in asymptomatic infections [1, 25, 26, 28]. As a result, an introduction of low virulent ASF, particularly in herds with concurrent systemic diseases, may go unnoticed, and identifying pigs to target for sampling may be difficult. Random sampling of apparently healthy pigs for testing may have greater utility for low virulence ASFV strains relative to moderate and virulent strains. However, more research is needed regarding active surveillance protocols for low virulence ASF.

The disease state and transmission parameters used in the ASF transmission model were largely derived from experimental studies [17, 23]. The applicability of these parameters to conditions in the field is uncertain. There is likely considerable variation in the field in factors such as stocking density, number of pigs per pen, ambient temperature, barn layout, and management practices that could impact transmission of ASFV within a barn. Differences in within-barn spread could impact the effectiveness of premovement active surveillance protocols, and a greater variety of outcomes may be observed in the field. While experimental conditions may resemble field conditions in some cases, in other cases they may differ. In particular, the pen size in the experiments was no more than 10 pigs, whereas the pen size used in the transmission model was 40 pigs. The effect of pen size on the transmission of ASF within and between pens is uncertain.

Since factors such as barn layout can impact ASF transmission, the applicability of these results to production types other than finishers (e.g., boar stud or gestation barns) is limited. Premovement active surveillance protocols for movement of animals or products out of a CA should be tailored to the barn layouts, production practices, and movement risk. Thus, more research is needed into premovement active surveillance protocols for other product and animal movements. The active and passive surveillance included in other ASF modeling studies have focused on mortality triggers for early detection or regular surveillance of premises located within CA-type zones established around confirmed positive farms [6, 7, 8, 9, 10, 11, 12]. The surveillance protocols in these analyses were generally “one-size-fits-all”, for example, testing up to five dead pigs per herd per week [7]. However, in this analysis, predicted outcomes were dependent on barn size and amount of mortality and morbidity during routine production, which suggests active surveillance protocols that vary according to barn characteristics may be more effective. Sykes et al. [13] modeled between premises the transmission of ASF in the US for different control scenarios, including permitted movement of animals or products from farms located within a CA [13]. The predicted outputs from the current analysis could be used to inform models between premises like the Sykes et al. [13] model, where premovement active surveillance was modeled more approximately.

Prioritizing pigs with clinical signs can be highly efficient at detecting ASFV in a barn, especially for moderately virulent strains. However, when the clinical signs are mild, sick pigs can be difficult to identify. The assumption made in this analysis was that pigs with mild to severe clinical signs could be identified with 85% probability, but there is considerable uncertainty surrounding this parameter. Even if pigs with mild clinical signs could be easily identified, collecting an antemortem specimen like blood from such pigs can be more difficult relative to collecting tissue specimens from dead pigs. Research regarding the probability of detection and ease of collection for both antemortem and postmortem specimens is needed. The value of testing pigs with clinical signs toward early detection of ASF also supports the need for proficiency and training in recognizing these animals.

Identifying and collecting samples from pigs with mild clinical signs are just examples of the complications when attempting to implement active surveillance protocols. Further complications involve factors such as distance of a farm from a diagnostic laboratory and the ability to receive a test result at or by the time of movement when sampling occurs just 24 hr prior. Additionally, while increasing the sample size may improve the predicted outcomes, increased sample sizes in tandem with the demand for just-in-time testing may not be compatible with laboratory capacity or ability.

Overall, there is a great deal of complexity surrounding the design of premovement active surveillance protocols for sending finishing pigs to a harvest facility from a CA during an outbreak of ASF. This analysis demonstrates how ASFV strain, farm size, and farm performance can all impact the risk of moving infectious pigs. For an effective outbreak response, control measures such as enhanced biosecurity, quarantines, and epidemiologic tracing should be performed in addition to premovement active surveillance. A multipronged approach to outbreak management is more robust than dependence on any one response measure. Navigating and resolving the complexity around premovement active surveillance and its place within a wider outbreak response are best done through multidisciplinary teams of diverse stakeholders including industry practitioners, state and federal regulators, and subject matter experts.

## 5. Conclusions

The effectiveness of active surveillance protocols for moving finishing pigs to a harvest facility out of a CA during an outbreak of ASF was dependent on numerous factors including sample size, sampling prioritization, ASFV strain, farm size, and amount of routine mortality and morbidity in a barn. Targeting sick and dead pigs for sampling was the most effective practice for increasing the probability of detection and reducing the number of infectious pigs at the time of movement when the virus went undetected. Increasing the sample size generally improved performance, though gains were minimal to nonexistent once the sample size exceeded the total mortality and morbidity in the barn. The probability of detection was marginally higher when all available mortality and morbidity were sampled across 2 days prior to movement as compared to 1 day. When the sample size was less than the amount of mortality and morbidity in the barn, taking all the samples 1 day before movement was more effective than splitting the samples across the 2 days prior. There was some evidence that sampling pigs with clinical signs first, then dead pigs, followed by randomly sampling living pigs would be most effective for moderately virulent strains. However, in general, there was little difference in the results across sampling prioritization schemes that included sick and dead pig targeting. The higher totals of mortality and morbidity during routine production associated with larger barn sizes and poor performance led to lower probabilities of detection and higher numbers of infectious undetected pigs, which suggests that higher sample sizes may be needed for large barns.

## Figures and Tables

**Table 1:** Within-barn ASFV transmission model input parameters for the moderately virulent ASFV strain scenario.

Parameter	Distribution/value^a^	References
Latent period length	Gamma (shape = 13.30, scale = 0.34); mean = 4.50 days, s.d. = 1.23 days	[12]
Infectious period length for recovered	Gamma (shape = 55.42, scale = 0.80); mean = 44.06 days, s.d. = 5.92 days	[12]
Infectious period length for dead	Gamma (shape = 9.63, scale = 0.86); mean = 8.30 days, s.d. = 2.68 days	[12]
Time to onset of clinical signs	Gamma (shape = 26.26, scale = 0.21); mean = 5.62 days, s.d. = 1.10 days	[12]
Duration of clinical signs	Gamma (shape = 3.42, scale = 3.20); mean = 10.94 days, s.d. = 5.92 days	[12]
Time pig is viremic relative to onset of infectiousness	Normal (mean = −0.82, s.d. = 0.82) days	[17]
Fraction of pigs dying due to ASF	0.40	[12]
Within-pen transmission rate (*β*_*w*_)	Beta-PERT (min = 1.00, mode = 1.64, max = 2.74) contacts per day	[12]
Between-pen transmission rate (*β*_*b*_)	Beta-PERT (min = 0.10, mode = 0.30, max = 0.50) contacts per day	[15]
Proportion of between-pen spread due to distance-independent pathways (*θ*)	0.05	Expert opinion [18, 19]
Between-room transmission rate (*β*_*room*_)	Beta-PERT (min = 0.001, mode = 0.05, max = 0.15) contacts per day	Expert opinion [9]

^a^See the supplementary materials for definitions of the gamma, normal, and beta-PERT distributions.

**Table 2:** Within-barn ASFV transmission model input parameters for the highly virulent ASFV strain scenario.

Parameter	Distribution/value^a^	References
Latent period length	Gamma (shape = 31.87, scale = 0.12); mean = 3.87 days, s.d. = 0.69 days	[20]
Infectious period length	Gamma (shape = 8.88, scale = 0.51); mean = 4.51 days, s.d. = 1.51 days	[20]
Time to onset of clinical signs	Gamma (shape = 44.10, scale = 0.09); mean = 4.13 days, s.d. = 0.62 days	[20]
Time pig is viremic relative to onset of infectiousness	Normal (mean = −1.15, s.d. = 0.18) days	[20]
Fraction of pigs dying due to ASF	1.00	[20]
Within-pen transmission rate (*β*_*w*_)	Beta-PERT (min = 0.96, mode = 2.62, max = 5.61) contacts per day	[16]
Between-pen transmission rate (*β*_*b*_)	Beta-PERT (min = 0.31, mode = 0.99, max = 1.98) contacts per day	[16]
Proportion of between-pen spread due to distance-independent pathways (*θ*)	0.05	Expert opinion [18, 19]
Between-room transmission rate (*β*_*room*_)	Beta-PERT (min = 0.001, mode = 0.05, max = 0.15) contacts per day	Expert opinion [9]

^a^See the supplementary materials for definitions of the gamma, normal, and beta-PERT distributions.

**Table 3:** Key input parameters for the active surveillance model.

Parameter	Distribution/value	References
Weekly proportion of pigs that die during routine production	Average: mean 0.003; 2.5 and 97.5 quantiles (0.000, 0.014) High: mean 0.014; 2.5 and 97.5 quantiles (0.011, 0.022)	Weekly mortality data from 248 finisher pig herds

Daily proportion of pigs with clinical signs during routine production	Average: beta-PERT (min = 0.0025, mode = 0.005, max = 0.04) High: beta-PERT (min = 0.01, mode = 0.02, max = 0.045)	Expert opinion [12]

Probability of observing a pig with clinical signs	0.85	Expert opinion (ASF Stakeholder Workgroup, personal communication, November 2022)

qPCR sensitivity	0.95	[24]

Mortality trigger threshold	Greater than five dead per 1,000 on a single day	[12]

**Table 4:** Predicted premovement active surveillance protocol outcomes for 1,200 head barns with average routine mortality and morbidity assuming a random time of virus exposure between 1 and 30 days prior to movement.

Active surveillance protocol	ASFV strain scenario	Sampling prioritization			
		Dead then sick then apparently healthy	Sick then dead then apparently healthy	Dead up to five then sick then apparently healthy	Random live
Protocol A: Five individual pigs sampled 24 and 48 hr prior to movement (total of 10 samples)	Moderately virulent	0.70^a^ 1 (0, 8)^b,c^	0.70 1 (0, 8)	0.69 1 (0, 9)	0.28 23 (0, 118)
	Highly virulent	0.78 1 (0, 7)	0.76 1 (0, 9)	0.78 1 (0, 6)	0.51 9 (0, 56)

Protocol B: 10 individual pigs sampled 24 hr prior to movement	Moderately virulent	0.72 1 (0, 7)	0.73 1 (0, 7)	0.73 1 (0, 8)	0.28 22 (0, 116)
	Highly virulent	0.79 1 (0, 6)	0.78 1 (0, 7)	0.78 1 (0, 6)	0.53 9 (0, 57)

Protocol C: 15 individual pigs sampled 24 hr prior to movement	Moderately virulent	0.74 1 (0, 5)	0.75 1 (0, 5)	0.75 1 (0, 5)	0.34 18 (0, 108)
	Highly virulent	0.80 1 (0, 4)	0.81 1 (0, 5)	0.81 1 (0, 5)	0.55 8 (0, 51)

Protocol D: 15 individual pigs sampled 24 and 48 hr prior to movement (total of 30 samples)	Moderately virulent	0.78 1 (0, 3)	0.78 1 (0, 3)	0.77 1 (0, 3)	0.42 11 (0, 75)
	Highly virulent	0.83 0 (0, 1)	0.82 0 (0, 1)	0.83 0 (0, 1)	0.58 7 (0, 46)

Protocol E: 30 individual pigs sampled 24 hr prior to movement	Moderately virulent	0.76 1 (0, 4)	0.75 1 (0, 5)	0.75 1 (0, 5)	0.44 11 (0, 75)
	Highly virulent	0.80 1 (0, 5)	0.81 1 (0, 4)	0.81 1 (0, 5)	0.60 6 (0, 41)

Protocol F: 30 individual pigs sampled 24 and 48 hr prior to movement (total of 60 samples)	Moderately virulent	0.77 1 (0, 3)	0.78 1 (0, 2)	0.78 1 (0, 2)	0.52 6 (0, 41)
	Highly virulent	0.83 0 (0, 1)	0.83 0 (0, 1)	0.82 0 (0, 1)	0.62 4 (0, 30)

Protocol G: 60 individual pigs sampled d 24 hr prior to movement	Moderately virulent	0.75 1 (0, 4)	0.76 1 (0, 4)	0.76 1 (0, 4)	0.54 5 (0, 37)
	Highly virulent	0.81 1 (0, 4)	0.81 1 (0, 4)	0.82 1 (0, 4)	0.65 4 (0, 26)

Protocol H: 100 individual pigs sampled 24 hr prior to movement	Moderately virulent	0.76 1 (0, 4)	0.76 1 (0, 4)	0.77 1 (0, 5)	0.58 3 (0, 22)
	Highly virulent	0.81 1 (0, 3)	0.82 0 (0, 2)	0.81 1 (0, 4)	0.68 2 (0, 16)

^a^The probability of detecting ASFV in an infected finisher barn located in a CA prior to movement of pigs to a harvest facility; ^b^the mean number of infectious pigs at the time of movement (95% P.I.) in barns where ASFV is not detected prior to the movement of pigs; ^c^predicted outcomes estimated from 10,000 simulation iterations and based on qPCR with a test sensitivity of 0.95 on sample pools of five of the same specimen type.

**Table 5:** Predicted premovement active surveillance protocol outcomes for 4,800 head barns with high mortality and morbidity assuming a random time of virus exposure between 1 and 30 days prior to movement.

Active surveillance protocol	ASFV strain scenario	Sampling prioritization			
		Dead then sick then apparently healthy	Sick then dead then apparently healthy	Dead up to five then sick then apparently healthy	Random live
Protocol A: Five individual pigs sampled 24 and 48 hr prior to movement (total of 10 samples)	Moderately virulent	0.34^a^ 18 (0, 115)^b,c^	0.51 7 (0, 49)	0.35 19 (0, 123)	0.12 54 (0, 327)
	Highly virulent	0.62 5 (0, 34)	0.60 6 (0, 47)	0.60 5 (0, 34)	0.41 23 (0, 113)

Protocol B: 10 individual pigs sampled 24 hr prior to movement	Moderately virulent	0.39 16 (0, 105)	0.50 7 (0, 43)	0.46 10 (0, 72)	0.13 53 (0, 321)
	Highly virulent	0.63 5 (0, 31)	0.62 5 (0, 39)	0.62 5 (0, 32)	0.41 23 (0, 112)

Protocol C: 15 individual pigs sampled 24 hr prior to movement	Moderately virulent	0.48 9 (0, 59)	0.56 4 (0, 28)	0.53 5 (0, 37)	0.17 45 (0, 276)
	Highly virulent	0.66 3 (0, 21)	0.65 4 (0, 25)	0.65 4 (0, 23)	0.41 21 (0, 109)

Protocol D: 15 individual pigs sampled 24 and 48 hr prior to movement (total of 30 samples)	Moderately virulent	0.54 5 (0, 30)	0.61 3 (0, 15)	0.58 3 (0, 22)	0.25 31 (0, 198)
	Highly virulent	0.70 2 (0, 14)	0.69 2 (0, 14)	0.70 2 (0, 13)	0.46 19 (0, 107)

Protocol E: 30 individual pigs sampled 24 hr prior to movement	Moderately virulent	0.59 3 (0, 18)	0.63 2 (0, 12)	0.61 3 (0, 16)	0.25 31 (0, 201)
	Highly virulent	0.71 2 (0, 12)	0.71 2 (0, 12)	0.71 2 (0, 11)	0.45 17 (0, 99)

Protocol F: 30 individual pigs sampled 24 and 48 hr prior to movement (total of 60 samples)	Moderately virulent	0.64 2 (0, 12)	0.68 2 (0, 9)	0.66 2 (0, 10)	0.34 21 (0, 142)
	Highly virulent	0.74 1 (0, 8)	0.73 1 (0, 9)	0.74 1 (0, 8)	0.50 14 (0, 91)

Protocol G: 60 individual pigs sampled d 24 hr prior to movement	Moderately virulent	0.68 1 (0, 8)	0.69 1 (0, 7)	0.69 1 (0, 8)	0.35 19 (0, 130)
	Highly virulent	0.77 1 (0, 7)	0.76 1 (0, 7)	0.76 1 (0, 8)	0.52 13 (0, 88)

Protocol H: 100 individual pigs sampled 24 hr prior to movement	Moderately virulent	0.75 1 (0, 5)	0.75 1 (0, 5)	0.75 1 (0, 5)	0.43 12 (0, 83)
	Highly virulent	0.80 1 (0, 4)	0.80 1 (0, 5)	0.80 1 (0, 6)	0.56 9 (0, 67)

^a^The probability of detecting ASFV in an infected finisher barn located in a CA prior to movement of pigs to a harvest facility; ^b^the mean number of infectious pigs at the time of movement (95% P.I.) in barns where ASFV is not detected prior to the movement of pigs; ^c^predicted outcomes estimated from 10,000 simulation iterations and based on qPCR with a test sensitivity of 0.95 on sample pools of five of the same specimen type.

## Data Availability

The data used to support the findings of this study are included in the article or available from the sources referenced in the article.

## Supplementary Materials

Supplementary Materials A description of the probability distributions used in the within-barn ASFV transmission model. Figure S1. Prevalence of detectable pigs with ASFV in the subpopulations of dead pigs, pigs with clinical signs, and living pigs, from 0 to 20 days post virus exposure, for a barn size of 4,800, with average and poor performance, and the moderately and highly virulent strain scenarios.
