# Supplementary material for: Simulation of Premovement Active Surveillance Protocols for Moving Finishing Pigs to a Harvest Facility from a Control Area during an Outbreak of African Swine Fever in the United States
Source: Transbound Emerg Dis. 2024 Jul 15;2024:6657600. doi: 10.1155/2024/6657600 (PMC12017237; doi:10.1155/2024/6657600)
Supplement: Supplementary Materials — A description of the probability distributions used in the within-barn ASFV transmission model. Figure S1. Prevalence of detectable pigs with ASFV in the subpopulations of dead pigs, pigs with clinical signs, and living pigs, from 0 to 20 days post virus exposure, for a barn size of 4,800, with average and poor performance, and the moderately and highly virulent strain scenarios. [file 6657600.f1.docx]

**Supplementary Materials**

**Description of probability distributions used in the stochastic within-barn ASFV transmission model**

Gamma distribution

A random variable X has a gamma distribution with parameters α and θ when the probability density function (pdf) is

$$f\left( x \right)=\frac{1}{\Gamma(\alpha)\theta^{\alpha}}x^{\alpha-1}e^{-\frac{x}{\theta}}, x>0; \alpha>0, \theta>0,$$

where $\Gamma(\alpha)$ is called the gamma function and has form

$$\Gamma\left( \alpha\right)=\int_{0}^{\infty} x^{\alpha-1}e^{-\frac{x}{\theta}}dx$$

The parameter denoted by α is the shape and the parameter denoted by θ is the scale. The mean of X is αθ and the variance is $\alpha\beta^{2}$ (1).

Normal distribution

A random variable X has a normal distribution (also known as Gaussian) with parameters μ and σ when the pdf is

$$f\left( x \right)=\frac{1}{\sqrt{2\pi\sigma^{2}}}e^{-\frac{1}{2e^{2}}\left( x-\mu\right)^{2}}, x\mathbb{\in R;}\mu\mathbb{\in R,}\sigma\in\mathbb{R}^{+}$$

The parameter denoted by μ is the mean and σ is the standard deviation. The variance of X is $\sigma^{2}$ (1).

Beta-PERT distribution

The Beta-PERT distribution (where “PERT” is an acronym for “project evaluation and review techniques”) is a modification of the beta distribution commonly used in risk analysis. The parameters of the Beta-PERT distribution consist of a minimum value ${(y}_{min})$, mode ${(y}_{mode})$, and maximum value ${(y}_{max})$. A random variable X has a beta distribution when the pdf is

$$f\left( x \right)=K{(x-a)}^{\delta}{(b-x)}^{\tau}$$

where *K* is a constant, *a* and *b* are endpoint parameters to be specified, and $\delta$ and $\tau$ are exponent parameters to be specified (2). In the Beta-PERT approach, $a=y_{min}$and $b=y_{max}$. Furthermore, the standard deviation is assumed to be $(y_{max}-y_{min})/6$ and the mean (μ) is $(y_{min}+4y_{mode}+y_{max})/6$ (2). Given these restrictions, $\delta$ and $\tau$ can be determined by the following expressions,

$$\delta=\frac{(\mu-y_{min})(2y_{mode}-y_{min}-y_{max})}{(y_{mode}-\mu)(y_{max}-y_{min})}$$

$$\tau=\frac{\delta(y_{max}-\mu)}{(\mu-y_{min})}$$

**Prevalence of pigs with detectable ASFV over time post virus exposure in a 4800 head barn**


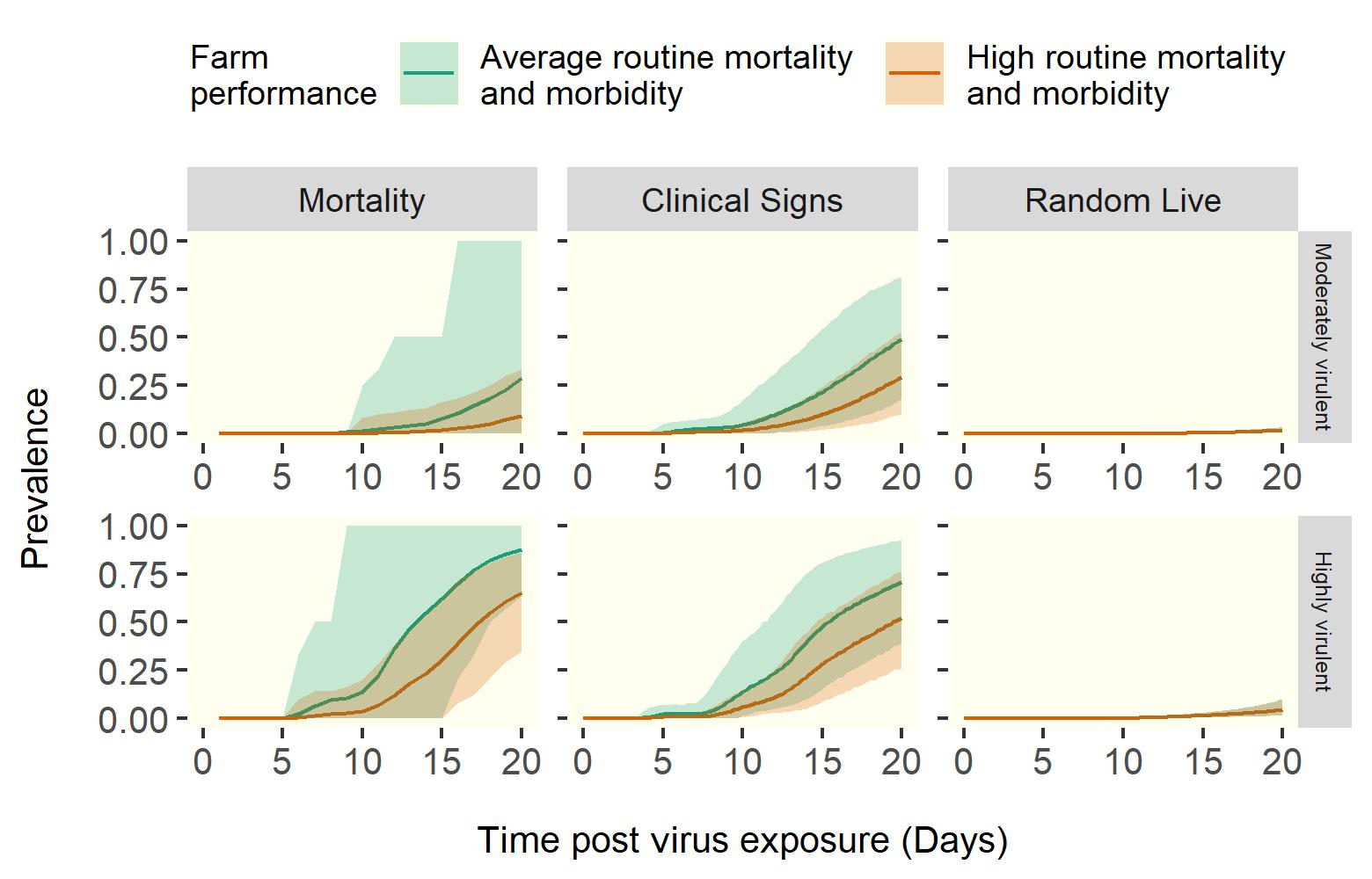


Figure S1. Mean (solid line) and 95% prediction intervals (P.I.) (shaded area) for the prevalence of pigs with detectable ASFV in different subpopulations for a 4800 head barn with average and high routine mortality and morbidity.

**References**

1. Rossi RJ. Mathematical statistics: an introduction to likelihood based inference. John Wiley & Sons; 2018.

2. MacCrimmon KR, Ryavec CA. An analytical study of the PERT assumptions. Operations Research. 1964;12(1):16–37.
